# Abscisic Acid Promotes Susceptibility to the Rice Leaf Blight Pathogen *Xanthomonas oryzae* pv *oryzae* by Suppressing Salicylic Acid-Mediated Defenses

**DOI:** 10.1371/journal.pone.0067413

**Published:** 2013-06-27

**Authors:** Jing Xu, Kris Audenaert, Monica Hofte, David De Vleesschauwer

**Affiliations:** 1 Laboratory of Phytopathology, Ghent University, Ghent, Belgium; 2 Faculty of Applied Bioscience Engineering, Ghent University College, Ghent, Belgium; University of Wisconsin-Milwaukee, United States of America

## Abstract

The plant hormone abscisic acid (ABA) is involved in a wide variety of plant processes, including the initiation of stress-adaptive responses to various environmental cues. Recently, ABA also emerged as a central factor in the regulation and integration of plant immune responses, although little is known about the underlying mechanisms. Aiming to advance our understanding of ABA-modulated disease resistance, we have analyzed the impact, dynamics and interrelationship of ABA and the classic defense hormone salicylic acid (SA) during progression of rice infection by the leaf blight pathogen *Xanthomonas oryzae* pv. *oryzae* (*Xoo*). Consistent with ABA negatively regulating resistance to *Xoo*, we found that exogenously administered ABA renders rice hypersusceptible to infection, whereas chemical and genetic disruption of ABA biosynthesis and signaling, respectively, led to enhanced *Xoo* resistance. In addition, we found successful *Xoo* infection to be associated with extensive reprogramming of ABA biosynthesis and response genes, suggesting that ABA functions as a virulence factor for *Xoo*. Interestingly, several lines of evidence indicate that this immune-suppressive effect of ABA is due at least in part to suppression of SA-mediated defenses that normally serve to limit pathogen growth. Resistance induced by the ABA biosynthesis inhibitor fluridone, however, appears to operate in a SA-independent manner and is likely due to induction of non-specific physiological stress. Collectively, our findings favor a scenario whereby virulent *Xoo* hijacks the rice ABA machinery to cause disease and highlight the importance of ABA and its crosstalk with SA in shaping the outcome of rice-*Xoo* interactions.

## Introduction

As sessile organisms, plants are continuously threatened by a suite of biotic and abiotic stress factors. Many of the defense mechanisms employed to counteract these stresses are controlled by an array of signal transduction pathways within which plant hormones function as key signaling molecules. Salicylic acid (SA), jasmonic acid (JA) and ethylene (ET) are the classic immunity hormones, while the importance of other small-molecule hormones including auxin, brassinosteroids (BR), gibberellic acid (GA), cytokinins (CK) and abscisic acid (ABA) is now gaining momentum [1]–[5]. Upon infection, plants produce a highly specific blend of hormonal alarm signals, resulting in the activation of disparate sets of attacker-specific immune responses [6]. SA, for instance, is commonly associated with defense against biotrophic pathogens, whereas necrotrophic pathogens are generally believed to be deterred by JA/ET-driven defenses [3].

Yet, rather than driving independent, linear routes of signal processing, hormones function within complex regulatory networks that connect the different pathways, enabling each to assist or antagonize the others. This interplay or so-called ‘crosstalk’ between individual hormones is thought to confer flexibility to the immune response, allowing the plant to adjust its inducible defense arsenal to the type of attacker encountered [7]. Exciting new developments, however, indicate that crosstalk may also allow successful pathogens to manipulate the plant’s defense signaling network for their own benefit by shutting down effective defenses [8]. A classic example reflecting this situation is the production by some *Pseudomonas syringae* strains of a phytotoxin called coronatine that structurally resembles JA derivatives, including JA-isoleucine [9]. Coronatine is actively secreted in the host and hyperactivates JA signaling, resulting in suppression of effectual SA-mediated defenses and increased disease susceptibility [10], [11].

Contrary to the relative wealth of information with respect to SA, JA and ET serving as defense regulators, the role of abscisic acid (ABA) in plant innate immunity is still poorly understood. Most comprehensively studied for its role in plant responses to environmental stresses, ABA has only recently emerged as a pivotal determinant in the outcome of plant-pathogen interactions [1], [12], [13]. In some interactions, ABA positively influences disease outcomes. For instance, ABA primes for callose deposition and thereby enhances basal defense against the powdery mildew fungus *Blumeria graminis* and the necrotrophic fungus *Alternaria brassicicola*, and also activates JA-mediated resistance against the oomycete *Pythium irregulare* [14], [15]. In addition, ABA is required for stomatal closure, which as part of the SA-mediated pre-invasion immune response, is a major barrier against bacterial invasion [16]. In most cases, however, ABA acts as a negative regulator of disease resistance with inhibition of ABA biosynthesis and/or signal transduction commonly resulting in enhanced disease resistance to a wide variety of bacterial, fungal and oomycete pathogens exhibiting distinct parasitic habits [17]–[25]. The importance of ABA in plant immunity is underscored by the ability of pathogens to either produce ABA themselves and/or to modify ABA biosynthesis and signaling in planta. In Arabidopsis, for instance, it was shown that *P. syringae* hijacks the ABA biosynthetic and response machinery to cause disease, indicating that ABA is a susceptibility factor for this bacterium [17]. Similarly, Jiang et al. (2010) reported transiently elevated ABA titers in rice plants attacked by the blast fungus *Magnaporthe oryzae* [26]. Current concepts suggest that this infection-induced ABA enables pathogens to tap into the plant’s defense signaling circuitry and interfere with host immunity. In support of this notion, there is ample evidence demonstrating the ability of ABA to interfere either directly or indirectly with the SA-JA-ET backbone of the plant defense circuitry [1], [3], [27], [28]. Additionally, ABA has been proposed to counteract GA-controlled defenses by promoting the stability of DELLA proteins that inhibit GA signaling [29], while exciting new molecular insights connect ABA also to CK-mediated stress responses [30]–[33].

Rice is one of the most important staple food crops worldwide, providing the bulk of the daily caloric intake for no less than 3 billion people living in tropical and subtropical Asia. However, despite its emergence as a pivotal model for studying innate immunity in monocotyledonous plants [34], studies addressing the role of plant hormones, and especially ABA, in the rice defensive machinery are scarce. In previous work, we have shown that ABA enhances basal resistance against the rice brown spot pathogen *Cochliobolus miyabeanus* by preventing the fungus from hijacking the ET pathway [35]. Interestingly, these ABA and ET-provoked effects are reverse of those against the blast fungus *M. oryzae*. In this pathosystem, ABA is thought to condition susceptibility via suppression of effectual ET- and SA-mediated defenses [26], [36]. In contrast, molecular information regarding the role of ABA in bacterial leaf blight (BLB) disease is still elusive. BLB, caused by the gram-negative bacterium *Xanthomonas oryzae* pv. *oryzae* (*Xoo*), is one of the most widespread and destructive rice diseases, causing annual yield losses up to 60% [37]. Aiming to further decipher the molecular underpinnings of ABA-modulated rice immunity, we sought to determine the impact, dynamics and inter-relationship of ABA with other hormones during progression of *Xoo* infection. Through genetic, physiological and pathological analyses, we show that ABA suppresses basal immunity of rice against virulent *Xoo* and likely functions as a virulence factor for the bacterium. Moreover, we demonstrate that ABA induces susceptibility of rice to *Xoo* by attenuating effectual SA defenses and provide evidence that this ABA-SA antagonism occurs downstream of SA biosynthesis, but upstream or at the level of the master defense regulators *OsNPR1* and *OsWRKY45*.

## Materials and Methods

### Plant Materials and Growth Conditions

Seeds of the *OsWRKY13*-OX [38], the *OsNPR1*-OX and the *OsNPR1* RNAi transgenics [39] and their respective wild-type lines Mudanjiang and Taipei were kindly provided by Dr. Wang (Huazhong Agricultural University, China) and Dr. He (Shanghai Institute for Biological Sciences, China), respectively. Rice *NahG* [40] and *OsMPK5* RNAi [41] seeds, and their parental line, *japonica* cultivar Nipponbare, were kind gifts from Dr. Yinong Yang (Pennsylvania State University, USA). *Indica* lines IRBB3 and IRBB13 were obtained from the International Rice Research Institute (courtesy of Casiana Vera-Cruz).

Unless stated otherwise, rice plants were grown in a hydroponic gnotobiotic system. Briefly, rice seeds were surface sterilized by agitation in 2% sodium hypochlorite for 20 min, rinsed three times with sterile demineralized water, and germinated for 5 days at 28°C on wet filter paper. Germinated seedlings were first sown in sterilized vermiculite supplemented with half-strength Hoagland solution. Two weeks later, the plants (3-leaf stage) were transferred to plastic containers containing modified Hoagland solution and grown for another three weeks under growth chamber conditions (28°C, relative humidity: 60%, 12/12 light regimen). For seed multiplication, plants were propagated in the greenhouse (30±4°C and 16 h photoperiod) and fertilized with 0.5% ammonium sulphate every two weeks until flowering.

### Pathogen Culture and Inoculation Assays

*Xanthomonas oryzae* pv. *oryzae* strain PXO99 (Philippine race 6) [42] was routinely grown on Sucrose Peptone Agar (SPA) medium at 28°C. For inoculation experiments, single colonies were transferred to liquid SP medium and grown for 48 h at 28°C. Plants were inoculated when 6 weeks olds by clipping the fifth and sixth stage leaves with scissors dipped in a solution of *Xoo* cells in water (1×10^9^ CFU.mL^−1^). Inoculated plants were kept in a dew chamber (≥92% relative humidity; 28±2°C) for 24 h and thereafter transferred to greenhouse conditions for disease development. Fourteen days after inoculation, disease severity was assessed by measuring the length of the water-soaked lesions. For bacterial growth analysis, inoculated leaves from three plants were pooled, ground up thoroughly using mortar and pestle and resuspended in 5 to 10 ml water. The leaf suspensions were diluted accordingly and plated on SPA. Plates were incubated at 28°C and colonies were counted within 2–3 days.

### Chemical Treatments

Stock solutions of SA (Sigma, Bornem, Belgium) were prepared directly in water, whereas fluridone (Fluka, Bornem, Belgium) and ABA (Duchefa, Schaarbeek, Belgium) were first dissolved in a few drops of methanol and ethanol, respectively. Equivalent volumes of both solvents were added to separate control treatments to ensure they did not interfere with the experiments. Fluridone was applied 6 days before *Xoo* inoculation by adding the compound to the modified Hoagland solution at a concentration of 0.4 µM. ABA and SA, on the other hand, were diluted in 0.02% (v/v) Tween 20 and applied as a foliar spray 72 h before inoculation. Control plants were sprayed evenly with 0.02% (v/v) Tween 20 only. For crosstalk experiments, fresh leaves from 6-week old rice seedlings were detached, cut into 3 cm pieces and subsequently incubated in the indicated hormone solutions for 8 h at 28°C. Leaf pieces from 13 plants were pooled and distributed randomly across the different treatments.

### RNA Extraction and Quantitative RT-PCR

Total leaf RNA was extracted using TRIZOL reagent (Invitrogen) and subsequently treated with Turbo DNase (Ambion) to remove genomic DNA contamination. First-strand cDNA was synthesized from 2 µg of total RNA using Multiscribe reverse transcriptase (Applied Biosystems) and random primers following the manufacturer’s instructions. Quantitative PCR amplifications were conducted in optical 96-well plates with the Mx3005P real-time PCR detection system (Stratagene), using Sybr Green master mix (Fermentas) to monitor dsDNA synthesis. The expression of each gene was assayed in duplicate in a total volume of 25 µL including a passive reference dye (ROX) according to the manufacturer’s instructions (Fermentas). The thermal profile used consisted of an initial denaturation step at 95°C for 10 min, followed by 40 cycles of 95°C for 15 s, 59°C for 30 s, and 72°C for 30 s. To verify amplification of one specific target cDNA, a melting-curve analysis was included according to the thermal profile suggested by the manufacturer (Stratagene). The amount of plant RNA in each sample was normalized using *eEF1α* (Eukaryotic elongation factor 1-alpha) as internal control [43]. Nucleotide sequences of all primers used are listed in Table S1. Group-wise comparison and statistical analysis of relative expression results was performed using Relative Expression Software Tool (*REST©)* [44].

## Results

### ABA Negatively Regulates Resistance to *Xoo*

**Figure 1:**
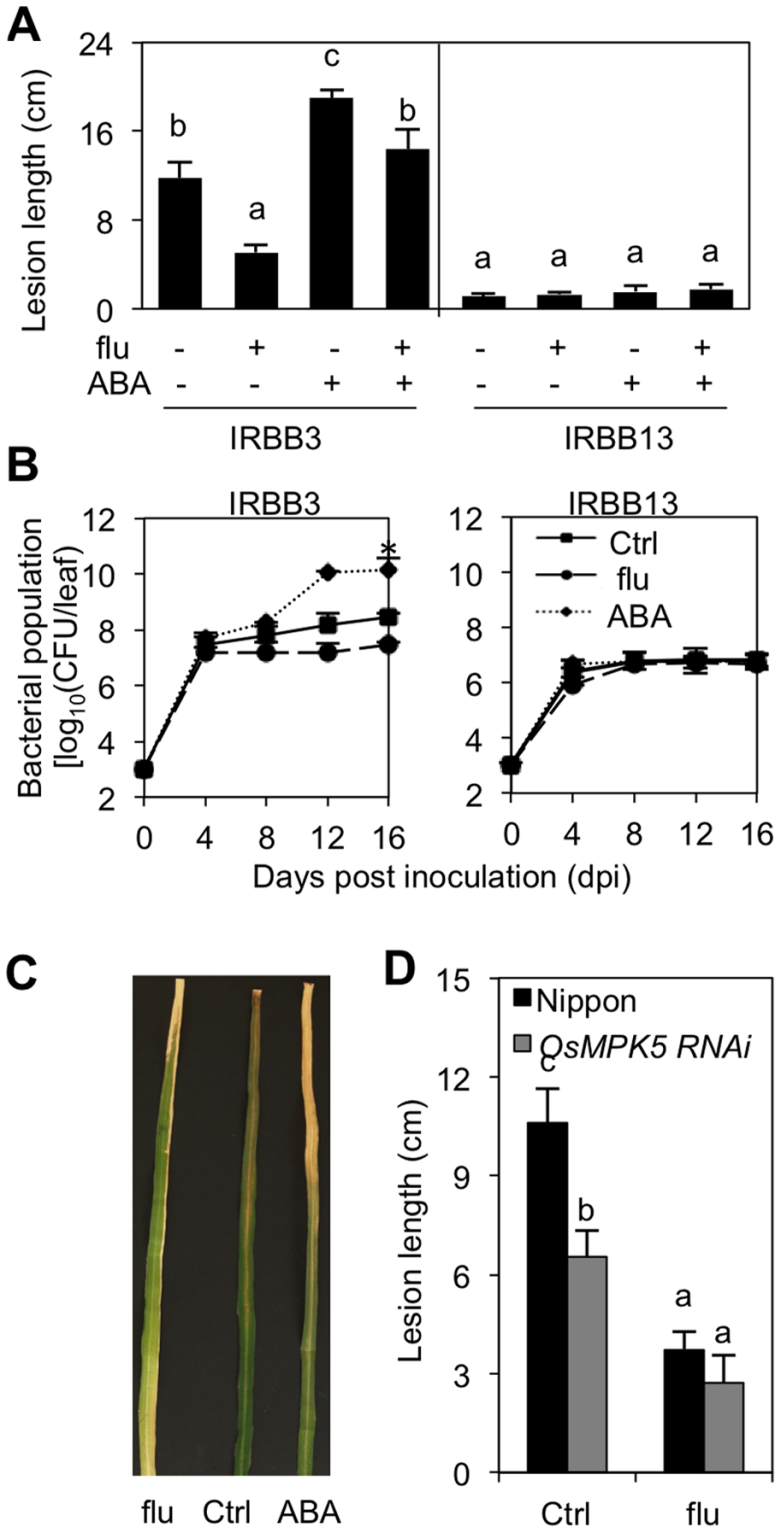
Effect of exogenous and endogenous abscisic acid (ABA) on bacterial leaf blight (BLB) development in rice. **(**A). Susceptible IRBB3 and resistant IRBB13 plants were pretreated with ABA (100 µM) and/or the ABA inhibitor fluridone (flu; 0.4 µM) for 3 and 6 days, respectively. Fifth and sixth stage leaves were inoculated with *Xanthomonas oryzae* pv. *oryzae* (*Xoo*) strain PXO99 using the standard leaf-clipping method. Fourteen days post inoculation (dpi), disease was evaluated by measuring the length of the water-soaked BLB lesions. Data are means ± SE of at least 10 plants. Different letters indicate statistically significant differences (Mann-Whitney: n ≥20; α = 0.05). (B). Effect of ABA (100 µM and fluridone (0.4 µM) on PXO99 titers in susceptible IRBB3 and resistant IRBB13. Data are means ± SE of three biological replicates. Asterisks indicate statistically significant differences compared to control treatments (LSD; n = 3; α = 0.05). (C). Symptom development on Ctrl, ABA or fluridone-pretreated IRBB3 leaves at 14 dpi. (D). Effect of fluridone (0.4 µM) on BLB development in *OsMPK5* RNAi and WT Nipponbare plants. Data are means ± SE of at least 10 plants. Different letters indicate statistically significant differences (Mann-Whitney: n ≥20; α = 0.05). All experiments were repeated at least twice with similar results.

In a first attempt to unravel the role of ABA in the rice-*Xoo* pathosystem, we examined the effect of exogenous hormone application on subsequent pathogen inoculation. To this end, leaves of 6-week-old *indica* cultivars IRBB3 and IRBB13 were sprayed until runoff with a 100 µM ABA solution and, three days later, inoculated with *Xoo* strain PXO99 using the leaf-clipping method [45]. PXO99 is virulent to IRBB3, but avirulent to IRBB13 which harbors the recessive *R* gene *xa13* [46]. In all bioassays, disease development was routinely monitored at 14 dpi by recording the length of the water-soaked lesions characteristic of leaf blight disease. As shown in Figures 1A and 1C, exogenous ABA application significantly lowered basal disease resistance in the susceptible IRBB3 background, with average lesions of 18 cm on ABA-treated plants compared to control, non-treated plants, which displayed average lesion lengths of 12 cm. In contrast, resistant IRBB13 seedlings inoculated with PXO99 displayed only marginal symptom development (lesions shorter than 1 cm) and ABA pretreatment appeared to have little or no effect in this background.

To further characterize the effect of ABA on *Xoo* immunity, we next assessed the impact of *in planta* ABA levels. Due to the lack of well-characterized ABA-deficient mutants in rice, a pharmacological approach was followed whereby hydroponically grown IRBB3 and IRBB13 plants were supplied for 6 days with the ABA biosynthesis inhibitor fluridone [47], and subsequently inoculated with PXO99. Corroborating our results with exogenous ABA, fluridone application substantially reduced disease severity in susceptible IRBB3, but failed to exert an additive effect on the already high levels of *Xoo* resistance in IRBB13 (Figure 1A). Importantly, fluridone had no significant effect on *in vitro* growth of PXO99 (data not shown), demonstrating the involvement of plant-mediated responses.

Bacterial growth analyses correlated well with lesion length developments (Figure 1B). At 16 dpi, PXO99 titers reached approximately 2 × 10^10 ^cfu/leaf in ABA-pretreated IRBB3, a greater than 100-fold increase compared to non-treated control IRBB3. In fluridone-treated IRBB3, however, PXO99 grew 10-fold less than in the controls with populations leveling off to fewer than 2 × 10^7^ cfu/leaf. In contrast, no significant differences between treatments could be observed in resistant IRBB13 where PXO99 populations reached approximately 8×10^6 ^cfu/leaf within 16 dpi. Together with the results from the lesion length measurements, these data strongly suggest that ABA suppresses basal immunity to *Xoo* and, hence, acts as a negative regulator of BLB resistance.

To substantiate this hypothesis, we quantified the level of basal and fluridone-inducible *Xoo* resistance in plants silenced for the MAP kinase gene *OsMPK5*. One of the better studied MAP kinases in rice, *OsMPK5* has been shown to function as a positive regulator of ABA signaling in rice [41]. Accordingly, *OsMPK5* RNAi plants are partially ABA-insensitive and display reduced expression of ABA-responsive genes [36]. As shown in Figure 1D and consistent with previous results [34], non-treated *OsMPK5* RNAi plants were significantly less susceptible to PXO99 than similarly treated wild-type plants, while fluridone application was equally effective in both genotypes, further confirming the negative impact of ABA on basal *Xoo* resistance.

### Temporal Dynamics of ABA Biosynthesis and Signaling in Response to *Xoo* Inoculation

**Figure 2:**
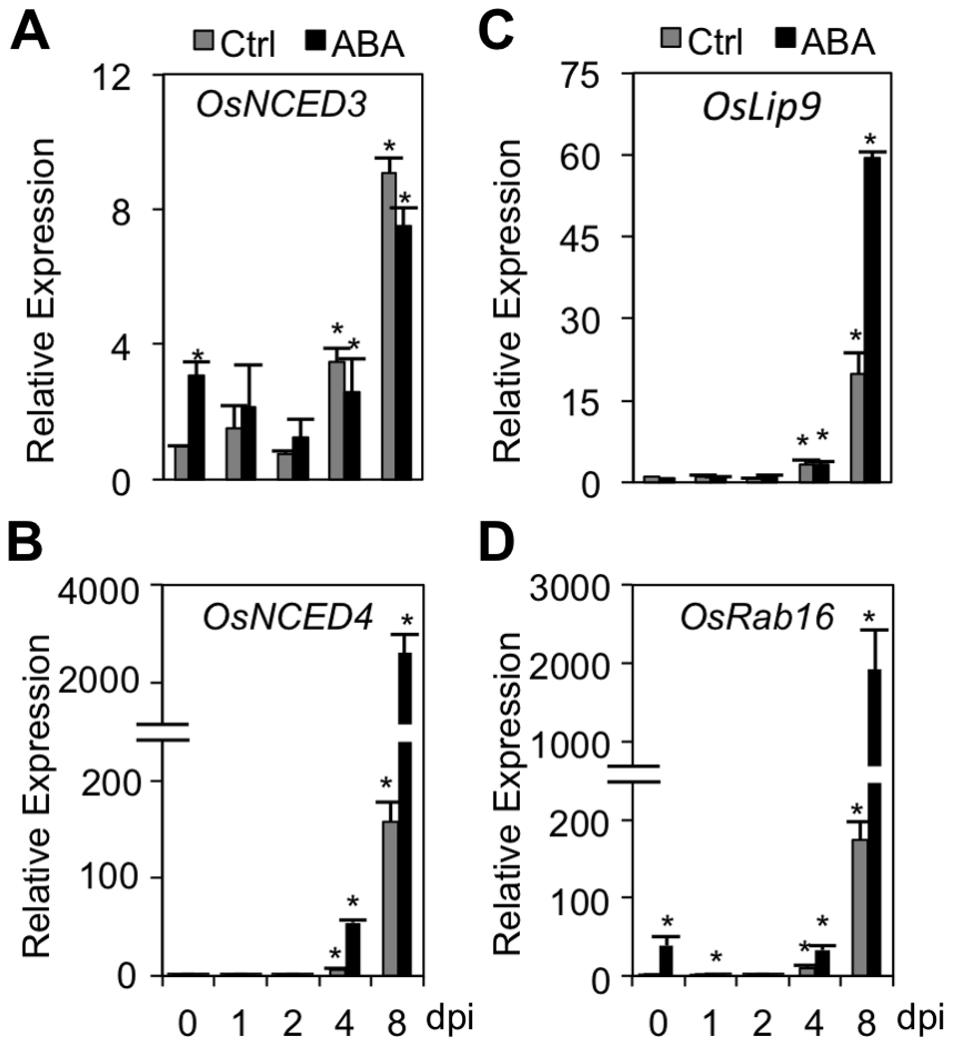
Dynamics of ABA pathway in response to virulent *Xanthomonas oryzae* pv. *oryzae* (*Xoo*) infection. (A) through (D). Effect of ABA pretreatment on ABA-biosynthesis (*OsNCED3*, *OsNCED4*) and ABA-responsive genes (*OsLip9* and *OsRab16*) in IRBB3 leaves inoculated with *Xoo* strain PXO99. For details on ABA pretreatment and *Xoo* inoculation, see legend to Figure 1. Transcript levels were normalized using eukaryotic elongation factor *eEF1α* as an internal reference and, for each treatment, expressed relative to the normalized expression levels in mock-inoculated control plants at the appropriate time point. Data are means ± SD of two technical and two biological replicates from a representative experiment, each biological replicate representing a pooled sample from 3 individual plants. Two sets of independent experiments were carried out with similar results. Asterisks indicate statistically significant differences per treatment compared to either control (0 dpi) or mock-treated samples (1, 2, 4 and 8 dpi).

To gain more insight into the mechanism(s) of ABA-induced *Xoo* susceptibility, we monitored the steady-state mRNA levels of several ABA biosynthetic and ABA responsive genes in control and ABA-pretreated IRBB3 leaves at various times after inoculation with PXO99. As shown in Figures 2A–B, expression of the ABA biosynthetic genes *OsNCED3* and *OsNCED4* remained static at early time points but increased steadily from 4 dpi and peaked at 8 dpi at approximately 10 and 150 times the levels found in non-inoculated controls, respectively. Interestingly, transcription of the ABA-responsive genes *OsLip9* and *OsRab16* mirrored the profiles observed for *OsNCED3* and *OsNCED4*, these genes being strongly upregulated at 4 and 8 dpi. Comparing control and ABA-treated samples at 0 dpi, no major differences could be observed for both *OsNCED4* and *OsLip9*. Expression of *OsNCED3* and *OsRab16*, on the other hand, was significantly higher in ABA-treated samples compared to control plants. In a similar vein, ABA application strongly boosted the expression of *OsNCED4* and both ABA-responsive genes following *Xoo* attack, especially at 8 dpi (Figure 2B–D).

**Figure 3:**
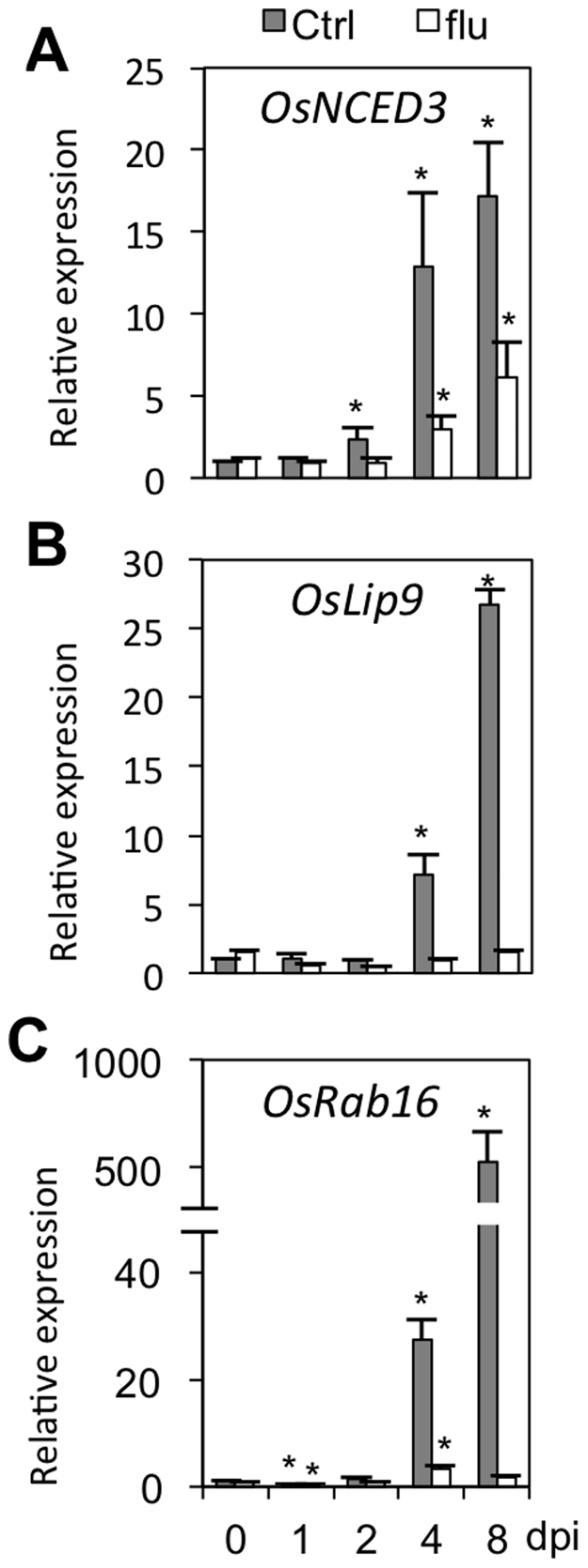
Cross-talk experiments demonstrating mutual antagonism between ABA and SA. IRBB3 leaf segments were incubated for 8 h in aequous solutions containing 50 µM ABA and/or 500 µM SA and subsequently tested for expression of the ABA responsive gene *OsLip9* and SA marker genes *OsNPR1* and *OsWRKY45*. Transcript levels were normalized using eukaryotic elongation factor *eEF1α* as an internal reference and for each treatment expressed relative to the normalized expression levels in non-treated control plants. Data are means ± SD of two technical and two biological replicates from a representative experiment, each biological replicate representing a pooled sample from 13 individual plants. The experiment was repeated once with similar results. Asterisks indicate statistically significant differences compared to control, non-treated samples.

In a set of parallel experiments, we also studied the expression profiles of *OsNCED3*, *OsLip9* and *OsRab16* in response to fluridone application. In line with abovementioned results, expression of these genes responded strongly to *Xoo* infection from 4 dpi onward (Figure 3A–C), whereas fluridone application strongly alleviated this pathogen-induced activation. Thus, ABA pretreatment boosts basal and/or pathogen-induced expression of ABA-responsive genes and enhances susceptibility to *Xoo*, whereas fluridone inhibits ABA-responsive gene expression and increases resistance to *Xoo*. When considered together, these data indicate that successful *Xoo* infection is associated with extensive reprogramming of ABA biosynthesis and ABA responsive genes. Moreover, in conjunction with earlier findings that ABA titers rise to a higher extent in compatible versus incompatible rice-*Xoo* interactions [48], these observations raise the possibility that virulent *Xoo* may hijack the rice ABA pathway to induce a state of susceptibility.

### ABA Negatively Regulates *Xoo* Resistance by Attenuating SA-mediated Defenses

**Figure 4:**
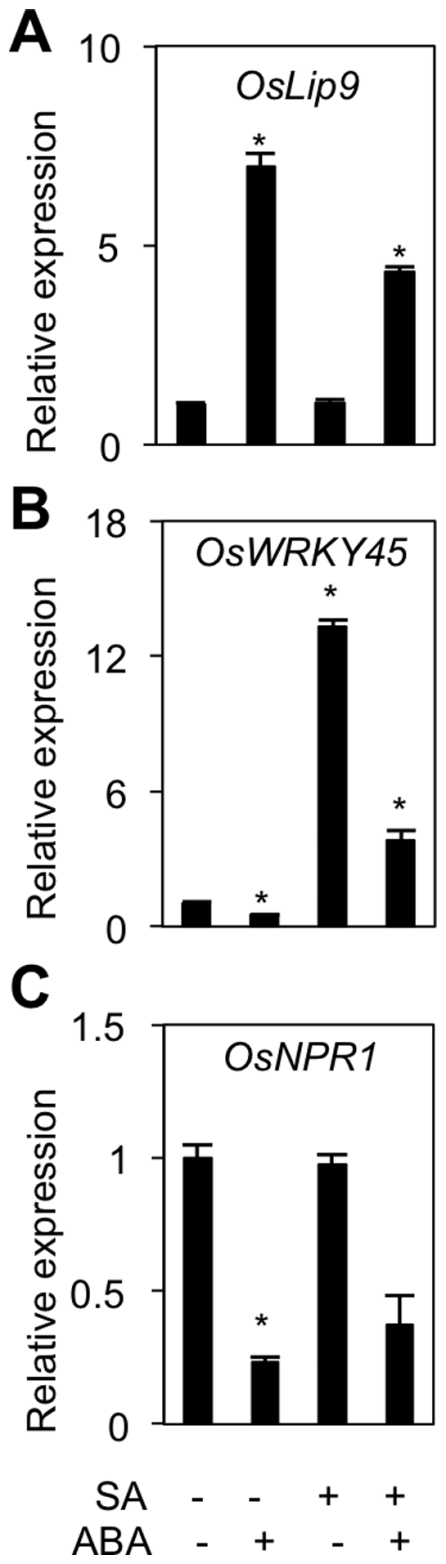
ABA counteracts SA-mediated defenses to *Xoo*. (A) through (C). Expression of SA marker genes *OsWRKY45, OsNPR1* and *OsWRKY13* in control (Ctrl) and ABA pretreated IRBB3 leaves inoculated with PXO99. Transcript levels were normalized using eukaryotic elongation factor eEF1α as an internal reference and for each treatment expressed relative to the normalized expression levels in mock-inoculated control plants at the appropriate time point. Data are means ± SD of two technical and two biological replicates from a representative experiment, each biological replicate representing a pooled sample from 3 individual plants. Asterisks indicate statistically significant differences per treatment compared to either control (0 dpi) or mock-treated samples (1, 2, 4 and 8 dpi). (D). Effect of single and combined pretreatment with ABA (100 µM) and/or SA (500 µM) on BLB development in susceptible IRBB3 plants. Lesions were measured 14 days after inoculation with PXO99. Data are means ± SE of at least 10 plants. Different letters indicate statistically significant differences (Mann-Whitney; n ≥20; α = 0.05) (E) and (F). Effect of exogenous ABA treatment (100 µM) on BLB development in *OsNPR1*-OX and *OsWRKY13*-OX lines and their respective WT Taipei and Mudanjiang. Data are means ± SE of at least 10 plants. Different letters indicate statistically significant differences (Mann-Whitney; n ≥20; α = 0.05). Repetition of experiments led to results similar to those shown.

In Arabidopsis, ABA has been repeatedly shown to negatively regulate plant disease resistance by antagonizing the SA signaling pathway [12], [24], [28]. Similarly, Jiang et al. (2010) [26] reported that ABA compromises resistance of rice to fungal blast disease by suppressing effective SA-mediated defense responses. To further confirm antagonistic crosstalk between ABA and SA in rice and expand the scope of the investigation, we assessed the effect of single and combined hormone treatments on the expression of ABA and SA marker genes. For this purpose, leaf blade segments of 6-week–old IRBB3 seedlings were incubated for 8 h in aqueous solutions of the respective hormones, and subsequently analyzed by quantitative RT-PCR. As shown in Figure 4A, single ABA treatment resulted in strong activation of the ABA marker gene *OsLip9*, while co-application of ABA with SA alleviated this ABA-induced *OsLip9* expression, indicating negative crosstalk in the direction of SA damping ABA action. However, consistent with bidirectional SA-ABA crosstalk, we also found ABA to impact the expression of both *OsNPR1* and *OsWRKY45*, two master regulatory proteins that control distinct branches of the SA signaling cascade in rice [39], [49]. Expression of *OsWRKY45* was activated in response to SA, whereas ABA suppressed both basal and SA-inducible *OsWRKY45* expression (Figure 4B). In contrast but consistent with previous reports on detached leaf segments [26], expression of *OsNPR1* was barely responsive to exogenous SA, though it was still markedly inhibited by ABA (Figure 4C).

**Figure 5:**
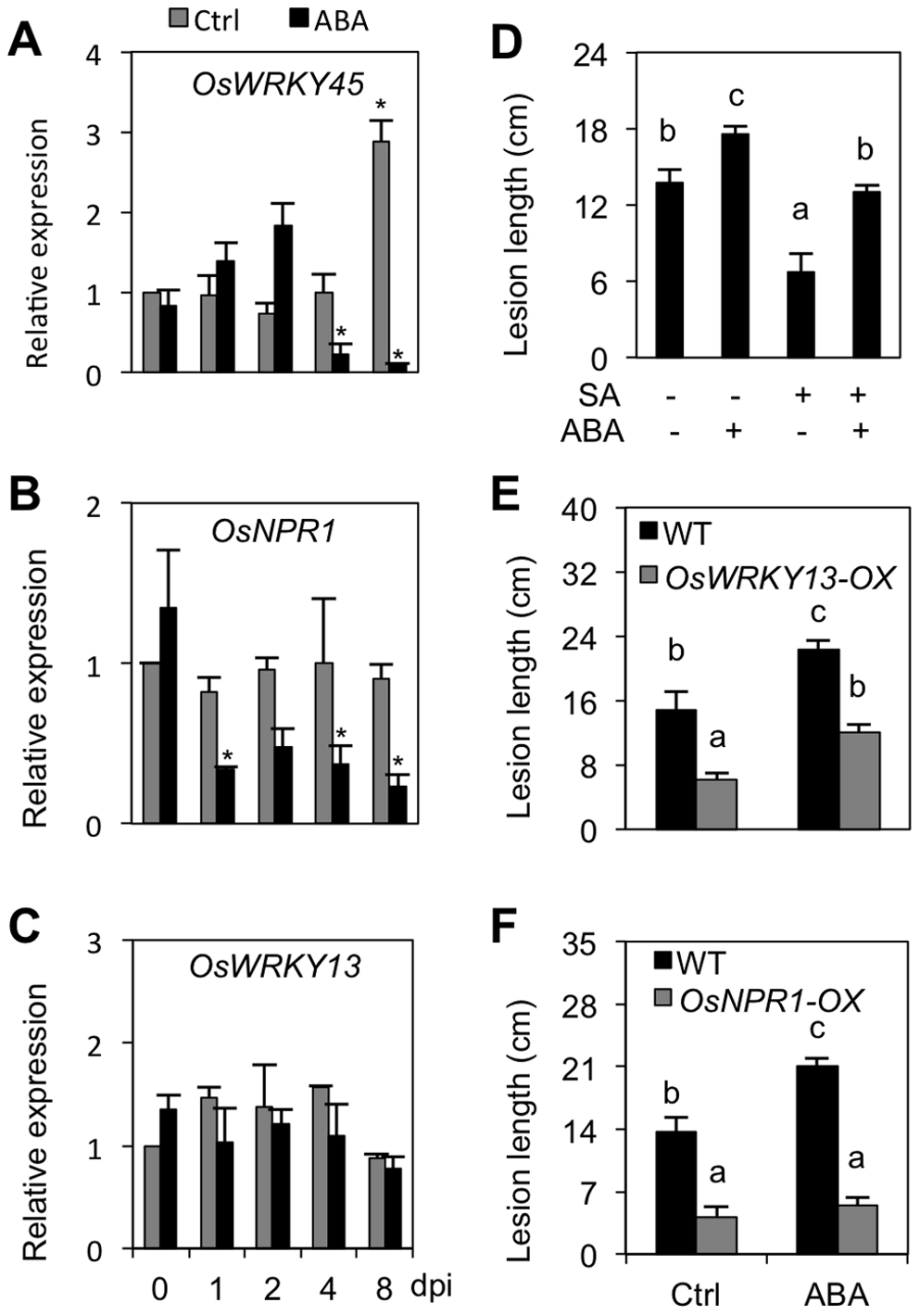
Fluridone suppresses pathogen-induced transcription of ABA biosynthesis and response genes. (A) through (C). Relative expression of ABA biosynthesis and responsive genes, *OsNCED3*, *OsLip9* and *OsRab16*, in control (Ctrl) and fluridone-pretreated (0.4 µM) IRBB3 leaves inoculated with PXO99. Transcript levels were normalized using eukaryotic elongation factor *eEF1α* as an internal reference and expressed relative to the normalized expression levels in mock-inoculated control plants at the appropriate time point. Data are means ± SD of two technical and two biological replicates from a representative experiment, each biological replicate representing a pooled sample from 3 individual plants. Two sets of independent experiments were carried out with similar results. Asterisks indicate statistically significant differences per treatment compared to either control (0 dpi) or mock-treated samples (1, 2, 4 and 8 dpi).

Having confirmed negative SA-ABA signal interactions in rice, we next sought to assess the significance of this antagonism in shaping the outcome of rice-*Xoo* interactions. To this end, leaves of 6-week-old IRBB3 were sprayed with 100 µM ABA and/or 500 µM SA and three days later, inoculated with virulent PXO99. As shown in Figure 5D, exogenous ABA treatment significantly enhanced disease susceptibility, whereas SA application rendered plants more resistant to subsequent PXO99 inoculation. Moreover, co-application with SA discounted the disease-promoting effect of single ABA treatments, suggesting that ABA may govern susceptibility to *Xoo* at least in part by suppressing effectual SA-mediated defenses.

To test this hypothesis, we monitored the temporal expression patterns of three SA regulatory genes in control and ABA-treated IRBB3 leaves following PXO99 infection. Besides *OsWRKY45* and *OsNPR1*, these genes included *OsWRKY13*, a well-characterized transcription factor gene functioning upstream of *OsWRKY45* and *OsNPR1* [38], [50]. Consistent with the expression profiles reported in other studies [38], [51]–[53], expression of *OsWRKY45* and *OsNPR1* responded only weakly to *Xoo* inoculation (Figure 5A–B). However, both genes were several-fold down-regulated in pathogen-inoculated leaves pretreated with ABA. Interestingly, ABA-mediated suppression of *OsWRKY45* was evident at 4 and 8 dpi only, which is in line with the upregulation of ABA biosynthesis and ABA signaling genes at these time points. In contrast, expression of *OsWRKY13* was not responsive to ABA treatment at any time point (Figure 5C), suggesting that ABA antagonizes SA-mediated *Xoo* resistance downstream of *OsWRKY13*. This notion was further supported by the different effects of ABA pretreatment on BLB development in transgenic rice lines overexpressing *OsNPR1* and *OsWRKY13*. Consistent with previous studies [38], [39], both *OsNPR1-*OX and *OsWRKY13-*OX lines exhibited increased resistance to *Xoo* compared to the respective wild-types (Figure 5E–F). However, while ABA application significantly promoted disease development in both WT and *OsWRKY13*-OX backgrounds, overexpressing *OsNPR1* fully blocked ABA-inducible *Xoo* susceptibility. Collectively, these data further confirm mutually antagonistic SA-ABA crosstalk during leaf blight infection and strengthen the hypothesis that ABA suppresses SA defenses downstream of *OsWRKY13* but upstream of *OsNPR1*.

### Fluridone-inducible *Xoo* Resistance is Independent of SA

**Figure 6:**
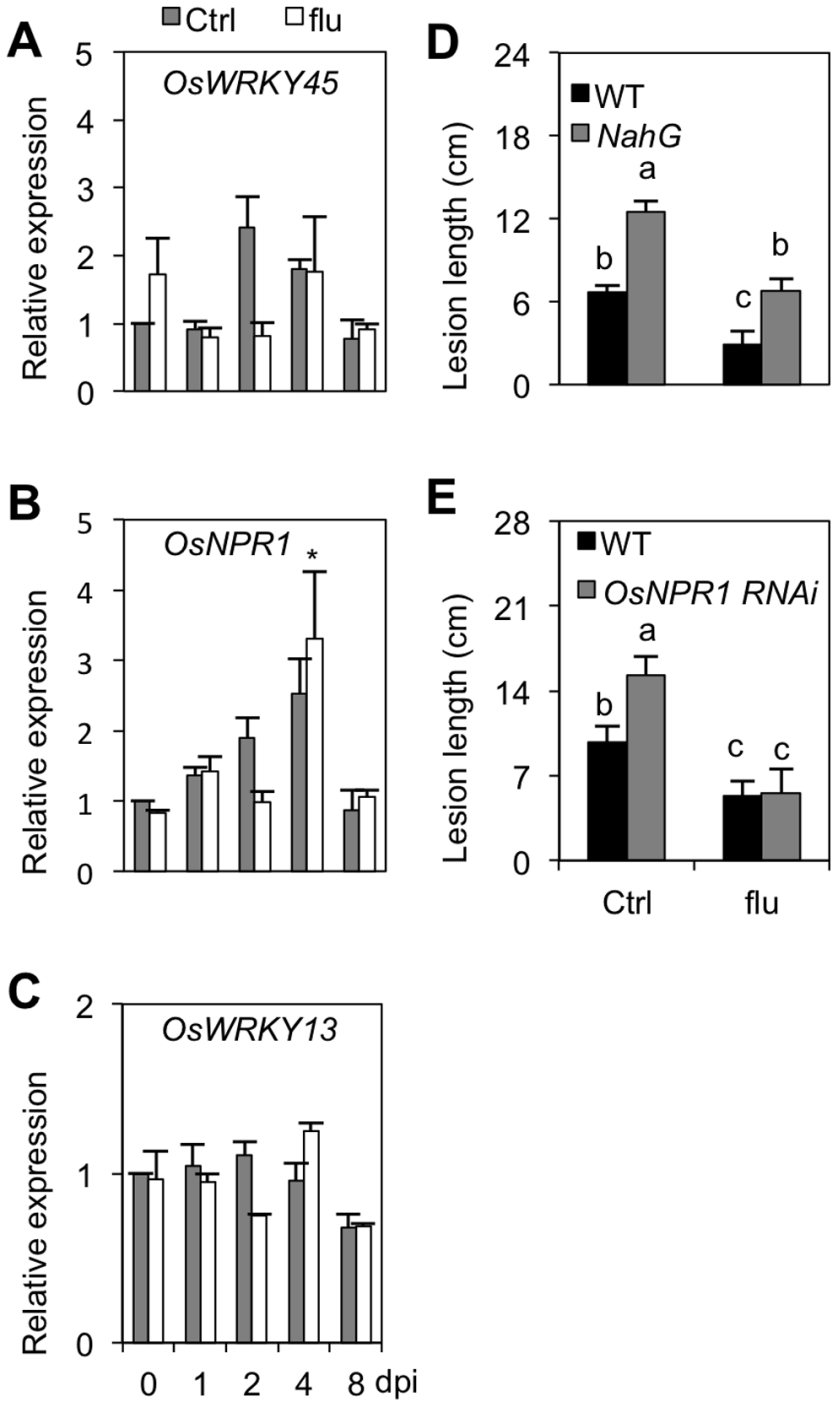
Fluridone induced *Xoo* resistance is independent of SA. (A) through (C). Transcript levels of the SA regulatory genes *OsWRKY45, OsNPR1* and *OsWRKY13* in control and fluridone-treated (0.4 µM) IRBB3 leaves inoculated with PXO99. Data are means ± SD of two technical and two biological replicates, each biological replicate representing a pooled sample from 3 individual plants. Asterisks indicate statistically significant differences per treatment compared to either control (0 dpi) or mock-treated samples (1, 2, 4 and 8 dpi). (D) and (E). Effect of fluridone (0.4 µM) on BLB development in *OsNPR1* RNAi and *NahG*-expressing lines and their respective WT Taipei and Nipponbare. Data are means ± SE of at least 10 plants. Different letters indicate statistically significant differences (Mann-Whitney; n ≥20; α = 0.05). Repetition of experiments led to results similar to those shown.

The observation that ABA induces *Xoo* susceptibility, at least in part, by antagonizing the SA pathway prompted us to check whether ABA-lowering fluridone induces resistance by de-repressing SA-mediated immune responses. To address this hypothesis, we initially checked the impact of fluridone application on the expression of the SA marker genes *OsWRKY45*, *OsNPR1* and *OsWRKY13* in IRBB3 leaves infected with PXO99. Consistent with Figures 5A–C, expression of *OsWRKY45*, *OsNPR1* and *OsWRKY13* showed little changes in response to PXO99 inoculation (Figure 6A–C). However, unlike the situation in ABA-treated leaves, no major and/or consistent changes in gene expression could be noticed between control and fluridone-treated samples, suggesting that fluridone-mediated resistance is not reliant on the SA pathway.

To further probe whether fluridone operates in an SA-independent manner, we quantified the level of fluridone-inducible resistance in both SA-non accumulating *NahG* and *OsNPR1* RNAi lines. As shown in Figure 6D, *NahG* plants were significantly more sensitive to pathogen attack than corresponding wild-type seedlings, demonstrating the importance of SA biosynthesis in basal resistance to *Xoo*. SA accumulation, however, did not appear to be a prerequisite for fluridone-inducible resistance, as fluridone application was equally effective in WT Nipponbare and *NahG* plants, causing an approximate 50% reduction in basal disease susceptibility in both genotypes. Similarly, fluridone triggered high levels of resistance in both WT Taipei and *OsNPR1* RNAi plants, indicating that, unlike ABA, fluridone functions independently of *OsNPR1* (Figure 6E).

## Discussion

Bacterial leaf blight (BLB), caused by the gram-negative bacterium *Xanthomonas oryzae* pv. *oryzae* (*Xoo*), is one of the most devastating rice diseases owing to its widespread distribution and high pathogenic variability. However, despite the accumulated wealth of genetic and molecular resources in rice and the identification of over 30 major resistance genes for BLB [37], surprisingly little is known about the hormone signaling pathways underpinning disease and resistance in the rice-*Xoo* pathosystem. Previously, Ding et al. [54] showed that auxin promotes susceptibility to *Xoo* through induced expression of cell wall-loosening expansins. In contrast, SA and JA act as positive regulators of immunity against *Xoo* [38], [39], [53], while GA and ET are reported to suppress BLB resistance through yet to be defined mechanisms [55], [56]. In adding to this list, our results uncover ABA as an additional negative regulator of rice-*Xoo* interactions. Moreover, our findings highlight the importance of bidirectional ABA-SA signal interactions in determining the outcome of rice-*Xoo* interactions and suggest that virulent strains of *Xoo* exploit ABA to subdue the rice innate immune system and promote disease development.

### ABA Negatively Regulates Rice Immunity to *Xoo*

Contrary to the well-characterized role of ABA in plant adaptive responses to abiotic stress [57], its contribution to plant disease resistance is relatively poorly understood, and even contentious. Whereas the majority of reports indicate that ABA suppresses pathogen defense responses [13], others have pinpointed a positive role of ABA in plant immunity [14], [35], [58]. Recent studies also found that the role of ABA in modulating disease resistance may depend not only on pathogen lifestyle but also on temporal and spatial conditions, indicating that complex nuanced mechanisms underlie ABA modulation of plant immunity [1], [12]. Under our experimental conditions, exogenous ABA application significantly increased rice susceptibility to virulent *Xoo* (Figure 1A), while lowering basal ABA levels by applying the ABA biosynthesis inhibitor fluridone or genetic disruption of ABA signaling in *OsMPK5* RNAi plants led to reduced disease development (Figure 1A–B). Similar to what was previously reported for the leaf blast fungus *M. oryzae* [26], [59], [60], ABA thus seems to act as a negative regulator of rice immunity to *Xoo*.

Interestingly, both exogenous ABA treatment and fluridone application failed to alter lesion length development and bacterial growth in IRBB13 plants carrying the recessive *R* gene *xa13*, suggesting that ABA predominantly affects basal defense responses against *Xoo*. However, care should be taken when interpreting these data. Recently, Mang et al. (2012) mechanistically connected ABA to *R* protein-mediated immunity by demonstrating that ABA deficiency in Arabidopsis promotes defense responses at high temperatures through enhancing the nuclear accumulation and activity of the resistance proteins *SNC1* and *RPS4* [61]. Consistent with this, exogenous ABA treatment was previously reported to compromise resistance to both virulent and avirulent blast fungus isolates, indicating that ABA negatively orchestrates both basal and *R* protein-mediated resistance against *M. oryzae* [26]. Taking these facts into account, it is not inconceivable that ABA may play a role in *Xoo* resistance governed by *R* genes other than *xa13*. Additional bio-assays using *Xoo* strains with different genetic backgrounds and rice lines carrying distinct types of *R* genes will aid in deciphering the role, if any, of ABA in regulating *R*-gene mediated resistance to *Xoo*.

The importance of ABA in determining pathological outcomes is underscored by the efforts pathogens undertake to tap into the host ABA biosynthesis and signaling infrastructure. Recent studies have demonstrated the direct manipulation of ABA biosynthesis and signaling by bacterial type III effectors as a virulence strategy for *P. syringae* and *X. campestris* pv. *campestris* [17], [25], [62], [63]. Moreover, in addition to modifying plant ABA biosynthesis, some phytopathogenic organisms, including the fungal pathogens *M. oryzae*, *Botrytis cinerea* and *Rhizoctonia solani*, are able to produce and secrete ABA themselves [64], [65]. Since there is no compelling evidence supporting the role of ABA in the physiology of these pathogens, it is likely that pathogens have evolved ABA biosynthetic machinery to trigger ABA signaling at infection sites and dampen plant immunity [12]. Previously, Liu et al [48] demonstrated that rice plants responding to *Xoo* attack accumulate substantial amounts of ABA from 4 dpi onwards, these levels being significantly higher in susceptible than in resistant plants. In view of these findings, the strong upregulation of ABA-biosynthesis and -responsive genes in control inoculated plants (Figure 2A–D; [48]), the disease-promoting effect of exogenously administered ABA (Figure 1A), and the positive correlation between bacterial growth and pathogen virulence on the one hand, and the amplitude of ABA-responsive gene expression on the other, strongly suggest that virulent *Xoo* may likewise co-opt the rice ABA machinery to promote bacterial growth and cause disease.

In this scenario, the identical bacterial densities observed during the first few days of inoculation in both compatible and incompatible rice-*Xoo* interactions (Figure 1B) are suggestive of an ABA-preceding interaction phase during which host and pathogen ‘battle’ for dominance. Depending on the outcome of this early interaction, *Xoo* strains may or may not be capable of hijacking the rice ABA pathway at late infection to achieve their full virulent potential. Although the molecular mechanisms underlying the early steps of rice-*Xoo* interactions are poorly resolved, recent transcriptome analyses and combined metabolite and hormone profiling increasingly implicate a coordinated range of hormone pathways. For instance, in resistant rice responding to virulent *Xoo*, both JA and ET signaling were found to be strongly activated within one hour after pathogen attack [66], whereas suppression of auxin and GA signaling seems to occur significantly later, i.e. between 12 hpi and 3 dpi [56], [67]. Together with our results, these data therefore suggest that temporally separated transient hormone changes play an important role in configuring the plant’s response to *Xoo* attack, with both host and pathogen trying to sequentially engage distinct hormone pathways in defined temporal windows.

### ABA Suppresses SA-mediated Defenses

Over the past decade, a multitude of mechanisms underpinning ABA’s broad and divergent impact on plant resistance responses have been identified. Besides interfering with pathogen-induced deposition of callose and modulating production of reactive oxygen species, ABA has been repeatedly shown to influence disease outcomes by interfering with other defense hormones [1], [12]. For instance, antagonistic or synergistic interactions between ABA and JA/ET are well known to play a pivotal role in numerous host-microbe interactions [27], [35], [36], [58], [68]. In addition, ABA has been proposed to antagonize SA-mediated signaling to regulate defense responses in tomato and Arabidopsis, where it affects both SA biosynthesis and signaling [24], [62]. In a similar vein, ABA enhances susceptibility of rice to *M. oryzae* by suppressing SA-regulated defenses [26]. Interestingly, several lines of evidence suggest that negative ABA-SA crosstalk also underpins the disease-promoting effect of ABA during rice-*Xoo* interactions. First, lesions caused by *Xoo* were more severe on SA-deficient *NahG* plants (Figure 6D), whereas topical application of SA or ectopic expression of the SA regulatory genes *OsNPR1* and *OsWRKY13* resulted in enhanced resistance (Figure 5D-F), tagging SA as a positive regulator of BLB resistance. Moreover, ABA not only antagonized SA-responsive gene expression in detached leaf assays but also down-regulated the transcription of SA regulatory genes during rice-*Xoo* interactions (Figure 4A–C and 5A–C) and, accordingly, attenuated SA-inducible pathogen resistance (Figure 5D). Finally and consistent with reciprocal antagonism in the direction of SA damping ABA action, we found SA to alleviate ABA-triggered effects on both marker gene expression and pathogen resistance (Figure 4A–C and 5D). When considered together, these data favor a scenario whereby mutually antagonistic ABA-SA crosstalk plays a central role in shaping the outcome of rice-*Xoo* interactions.

Interestingly, our data also infer that ABA antagonizes the SA signaling pathway downstream of *OsWRKY13* but upstream of *OsNPR1,* as overexpression of *OsNPR1* but not *OsWRKY13* abolished the negative impact of ABA on BLB resistance (Figure 5E–F). Potential target sites for ABA-mediated suppression of SA action include the transcription factors *OsWRKY71* and *OsWRKY24*, both of which function as transcriptional activators of SA signaling and are differentially expressed in response to *OsWRKY13* overexpression and/or ABA treatment [38], [69]. Alternatively, ABA may activate negative regulators of SA-responsive gene expression that either inhibit or out-compete positive regulators. Recently, Yasuda et al. (2008) identified multiple nodes of confluence between the SA and ABA signaling pathways in Arabidopsis [24]. Exploring whether similar crosstalk mechanisms are operative in rice is a major challenge for future research.

### Fluridone-inducible *Xoo* Resistance Functions Independently of SA

In higher plants, endogenous ABA is synthesized predominantly from zeaxanthin, which is an important intermediate in the carotenoid-biosynthesis pathway [70]. Fluridone is a herbicide that is widely used in ABA-related research because of its ability to block carotenoid synthesis, thus reducing ABA precursor pools. Based on the finding that ABA suppresses resistance to *Xoo* resistance by antagonizing SA defenses and given the strong negative effect of fluridone treatment on bacterial growth and pathogen-induced expression of ABA biosynthesis and response genes (Figure 1A–B and 3A–C), we initially hypothesized fluridone to enhance resistance to *Xoo* by de-repressing the SA pathway. Surprisingly, however, we failed to observe any significant or reproducible differences in SA-responsive gene expression between control and fluridone-treated plants (Figure 6A–C). Moreover, fluridone triggered wild-type levels of resistance in both *OsNPR1* RNAi and SA-deficient *NahG* seedlings, indicating that fluridone-inducible resistance requires neither SA biosynthesis nor SA action (Figure 6D–E). Although relatively little is known about the mechanism(s) of fluridone-mediated pathogen resistance, a few studies point to some possibilities. For example, Achuo et al. (2003) reported that micromolar concentrations of fluridone induced resistance of tomato against *Botrytis cinerea* without disturbing the plant ABA pool [71]. This result could be explained by assuming that fluridone caused some sort of physiological stress, the response to which resulted in disease resistance. Supporting this hypothesis, fluridone and norflurazone, another inhibitor of ABA biosynthesis, have been shown before to provoke physiological stress in plants through photobleaching of chlorophyll, a phenomenon also observed in this study (Figure 1C) [72]. Considering the strong impact of abiotic stress factors on plant immunity and the complex interplay between biotic and abiotic stress-response signaling pathways [57], [73], it is not unlikely that stress due to mild doses of photobleaching fluridone should result in disease resistance. Therefore, we propose that fluridone-mediated resistance to *Xoo* does not derive primarily from lowering ABA content and resultant activation of SA-mediated defenses, but rather is due to induction of non-specific physiological stress.

### Conclusions

**Figure 7:**
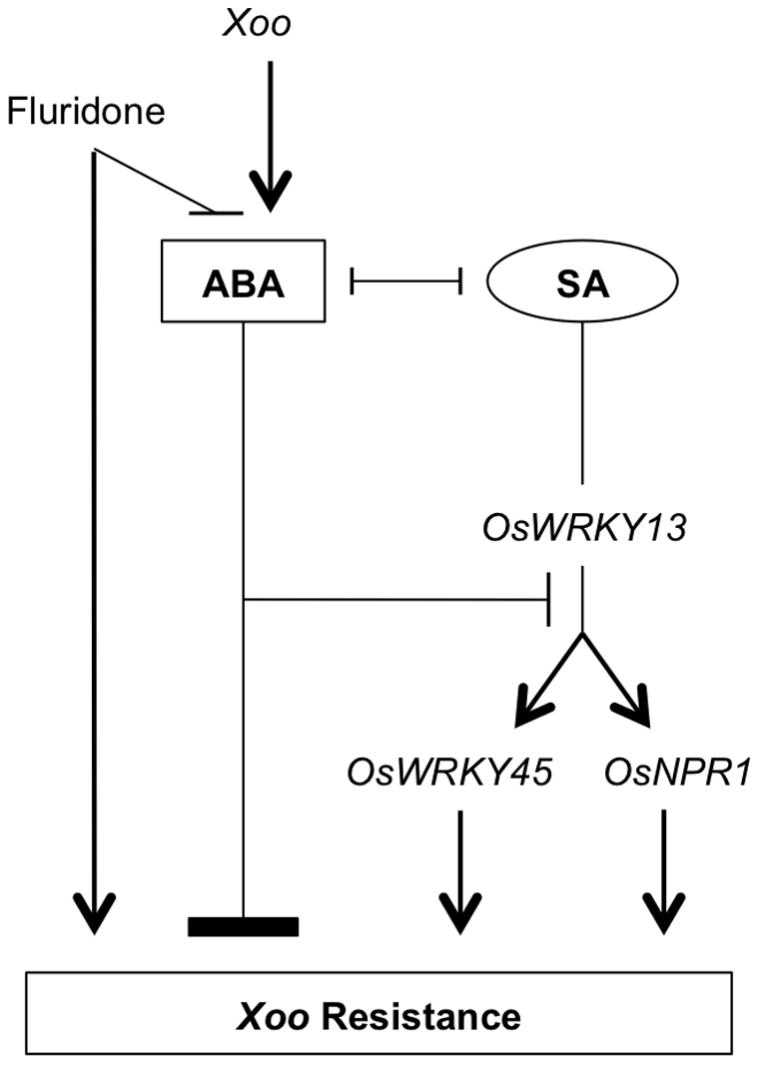
Model illustrating how dynamic interplay between ABA and SA molds innate immunity of rice against the BLB pathogen *Xoo*. Sharp arrows represent stimulatory effects, blunt arrows depict antagonistic interactions.

In conclusion, our results favor a model whereby ABA and its interaction with the SA pathway play central roles in orchestrating immunity of rice against the BLB pathogen *Xoo* (Figure 7). We propose that ABA acts as a virulence factor for *Xoo* by antagonizing effectual SA-mediated defenses downstream of the master regulator *OsWRKY13* but upstream of *OsNPR1*. In contrast, application of the ABA-lowering herbicide fluridone was found to trigger an SA-independent type of resistance. While bidirectional SA-ABA crosstalk may provide rice with a powerful potential to tailor its immune response to different types of attackers, our results suggest that virulent *Xoo* bacteria have evolved sophisticated strategies to manipulate ABA-SA interplay for their own benefit, redirecting the host immune response in favour of disease.

## Supporting Information

Table S1 **Sequences of qRT-PCR primers used in this study.**
(DOCX)
