# Correction: Abscisic Acid Promotes Susceptibility to the Rice Leaf Blight Pathogen *Xanthomonas oryzae* pv *oryzae* by Suppressing Salicylic Acid-Mediated Defenses

**DOI:** 10.1371/annotation/659105c2-8364-4cc7-94e7-66620370637a

**Published:** 2013-07-30

**Authors:** Jing Xu, Kris Audenaert, Monica Hofte, David De Vleesschauwer

The legends for Figures 3, 4 and 5 were accidentally switched. The legend appearing with the title and image of Figure 3 belongs with the title and image of Figure 4, the legend appearing with the title and image of Figure 4 belongs with the title and image of Figure 5, and the legend appearing with the title and image of Figure 5 belongs with the title and image of Figure 3. The titles and images are in correct order. 

