# Supplementary material for: Abscisic Acid Promotes Susceptibility to the Rice Leaf Blight Pathogen Xanthomonas oryzae pv oryzae by Suppressing Salicylic Acid-Mediated Defenses
Source: PLoS One. 2013 Jun 27;8(6):e67413. doi: 10.1371/journal.pone.0067413 (PMC3694875; doi:10.1371/journal.pone.0067413)
Supplement: Table S1 — Sequences of qRT-PCR primers used in this study. (DOCX) [file pone.0067413.s001.docx]

**Supplemental Table S1. Sequences of qRT-PCR primers used in this study**

| Gene name | Locus | Forward primer | Reverse primer |
| --- | --- | --- | --- |
| *eEF1a* | LOC_Os03g08020.1 | TTTCACTCTTGGTGTGAAGCAGAT | GACTTCCTTCACGATTTCATCGTAA |
| *OsNCED3* | LOC_Os03g44380.1 | TACGGCTTCCACGGCACGTTC | AGAAACGTGGAGGTGTTCGATCG |
| *OsNCED4* | LOC_Os07g05940.1 | ATTGCACGGCACCTTCATTG | ACCGCTAAACTATTTCAACTCCCT |
| *OsLip9* | LOC_Os02g44870.1 | CGGCGGCCTCTTCGAGACAAC | TGCCAGATTGCCAGCCCGTC |
| *OsRab16* | LOC_Os01g50700.1 | CACGAGTTCAGGGATCTAGGC | AGTTGTCCATCCTCTCAAGCAA |
| *OsNPR1* | LOC_Os01g09800.1 | CACGCCTAAGCCTCGGATTA | TCAGTGAGCAGCATCCTGACTAG |
| *OsWRKY45* | LOC_Os05g25770.1 | GGACGCAGCAATCGTCCGGG | CGGAAGTAGGCCTTTGGGTGC |
| *OsWRKY13* | LOC_Os01g54600.1 | AGCTGCCACGCGAGCAAGTC | GTCCGTCAGCCACCGGCTCAG |
